# Clinical Outcomes of Stage 2 (Pivotal) Use of a Modified Keratoprosthesis Device (ORC-KPro) in Patients with End-stage Corneal Blindness

**DOI:** 10.18502/jovr.v19i3.13307

**Published:** 2024-09-16

**Authors:** Saeed Rahmani, Farid Karimian, Kiana Hassanpour, Mohammad-Reza Jafarinasab, Sepehr Feizi, Sare Safi, Mohammad Ali Javadi

**Affiliations:** ^1^Department of Optometry, School of Rehabilitation, Shahid Beheshti University of Medical Sciences, Tehran, Iran; ^2^Ophthalmic Research Center, Research Institute for Ophthalmology and Vision Science, Shahid Beheshti University of Medical Sciences, Tehran, Iran; ^3^Department of Ophthalmology, Labbafinejad Medical Center, Shahid Beheshti University of Medical Sciences, Tehran, Iran; ^4^Ocular Tissue Engineering Research Center, Research Institute for Ophthalmology and Vision Science, Shahid Beheshti University of Medical Sciences, Tehran, Iran; ^5^Ophthalmic Epidemiology Research Center, Research Institute for Ophthalmology and Vision Science, Shahid Beheshti University of Medical Sciences, Tehran, Iran; ^7^https://orcid.org/0000-0001-6330-4405; ^8^https://orcid.org/0000-0001-6758-7457

**Keywords:** Corneal Blindness, Corneal Transplantation, Keratoprosthesis, KPro, ORC-KPro

## Abstract

**Purpose:**

To investigate the short-term results and performance of a modified Boston keratoprosthesis device manufactured by the Ophthalmic Research Center (ORC-KPro) in patients with end-stage corneal blindness.

**Methods:**

This prospective interventional case series was conducted on patients with corneal blindness who were candidates for KPro. The inclusion criterion comprised patients with a best-corrected visual acuity (BCVA) of less than 20/200 in both eyes, in whom the main reason for vision loss was corneal pathology. The ORC-KPro was implanted using the method previously described for Boston KPro.

**Results:**

This study focused on 12 eyes of 12 patients with an average age of 45.9 ± 16.8 (range, 19 to 70) years. Eleven patients were male. The KPro indication was corneal blindness due to chemical burns in nine patients (75%) and failure of multiple previous corneal grafts in three patients (25%). Anatomical success was achieved in all patients. The preoperative BCVA was light perception (LP) in 10 eyes and hand motion in 1 eye. Except for one patient who was diagnosed with grade C proliferative vitreoretinopathy during the surgery, the vision of all other patients (91.6%) improved after surgery. The retroprosthetic membrane (RPM) was formed in two eyes (18.1%) after six months. Of the 12 patients, 10 (83.3%) were under treatment with two antiglaucoma medications before surgery. The intraocular pressure of three eyes (25%) was estimated to be high by tactile palpation; however, it decreased in two eyes to the acceptable range. One patient underwent retinal surgery due to total retinal detachment, and two patients (16.7%) underwent vitrectomy due to endophthalmitis.

**Conclusion:**

The current study showed that, in the short term, the use of ORC-KPro achieved favorable anatomical success in patients with corneal blindness. However, the functional success rate was limited by the low visual potential due to advanced glaucoma in most patients.

##  INTRODUCTION

Keratoprosthesis (KPro) or artificial cornea transplant is the treatment of choice for patients with end-stage corneal blindness.^[1]^ In this method, a transparent prosthetic device is applied instead of a cornea to restore the patient's vision.^[2]^ As a standard indication, KPro is recommended in patients with corrected binocular vision of < 20/200.^[3]^ Various types of KPro have been introduced and are currently available. Boston type 1 KPro is used in patients with acceptable ocular surface, while Boston type 2 KPro and osteo-odonto KPro are recommended in patients with a compromised ocular surface.^[4,5]^ Boston type 1 KPro is a modification of the Dohlman type KPro which was approved in 1992 by the US Food and Drug Administration (FDA) for patients with low vision.^[6]^ It is estimated that 20,000 KPros have been used worldwide until 2023.

Boston type 1 KPro consists of a front and a back plate. In more recent designs, the locking ring has also been modified. The front plate contains an optical PMMA (polymethyl methacrylate) cylinder. The round posterior plate was first made of PMMA but it was changed to titanium with a diameter of 8.5 mm. It has a large central hole for the optical cylinder and 8–16 peripheral smaller holes, providing nutrition for the transplanted cornea from the aqueous humor.^[1]^

Indications for type 1 KPro include patients with multiple failed corneal grafts and/or high-risk grafts such as extensive neovascularization, aniridia with limbal stem cell deficiency, recurrent herpes simplex keratitis, and corneal infections.^[2,7,8,9,10,11,12,13]^ In a meta-analysis featuring a five-year follow-up, Ahmed et al showed that the use of Boston type 1 KPro compared to re-graft had a higher chance of maintaining clear cornea and visual acuity better than 20/200.^[14]^ More recent studies have shown that Boston type 1 KPro can also be used in the earlier stages of graft failure with a higher rate of success compared to re-graft.^[3,7,15]^

In developing countries, Boston type 1 KPro faces limitations in terms of access and cost. In 2018, a modified version of type 1 KPro was designed and developed in the Ophthalmic Research Center (ORC) affiliated with Shahid Beheshti University of Medical Sciences, Tehran, Iran, and was called ORC-KPro. After the first stage (pilot study) of evaluation and modification in the design and components of the primary KPro, the limitations and advantages of this device were identified. In the second stage (pivotal), the study was performed to investigate the short-term results and performance of the ORC-KPro in patients with end-stage corneal blindness.

##  METHODS

This prospective interventional case series was designed as the pivotal stage for the clinical evaluation of ORC-KPro. Patients with corneal blindness who were candidates for KPro surgery were enrolled. The study adhered to the tenets of the Declaration of Helsinki and was approved by the Ethics Committee of the Ophthalmic Research Center of Shahid Beheshti University of Medical Sciences (Code of Ethics No. IR.SBMU.ORC.REC.1399.019).

Eligible patients presented a best-corrected visual acuity (BCVA) < 20/200 in both eyes and the main reason for their vision loss was corneal pathology. The exclusion criteria included severe dry eye, a severely compromised ocular surface, absence of conjunctival fornices and conjunctival keratinization, poorly controlled glaucoma, consent refusal for the use of the ORC-KPro, absence of potential for vision improvement due to lack of light perception, absence of the optic nerve function (flat VEP), retinal detachment, and low adherence to the follow-up.

### ORC- KPro Characteristics

**Figure 1:**
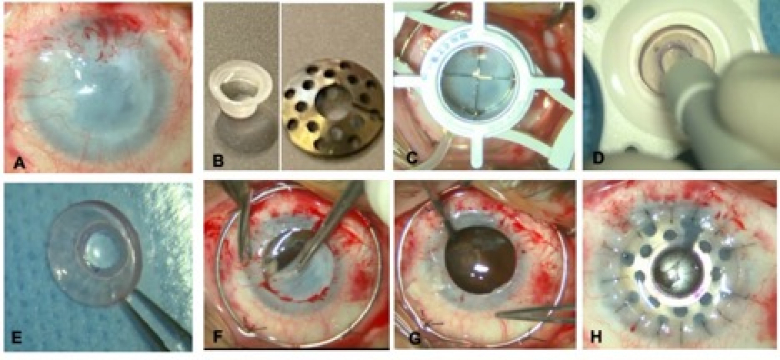
The surgical steps of KPro implantation in a patient with chemical burns (A). (B) The KPro produced by the Ophthalmic Research Center of Shahid Beheshti University of Medical Sciences, including the anterior cylinder (left image) and the titanium back plate on the right side. (C) The trephined (8.25 mm) cornea of the patient. (D) The central part of the punched corneal button punched with a 3-mm dermatomal punch. (E) The initial stages of assembling the KPro. First, the anterior plate is placed on the glue. The carrier cornea is placed in between, and in the next step, which is not shown in the picture, the posterior plate is added to these two parts. (F) The trephined cornea is gently removed from the eye. The Flieringa ring is sutured to the episclera at 3 or 4 points. (G) Anterior vitrectomy. In this patient, severe optic atrophy and posterior staphyloma are observed. (H) The KPro is seen on the patient's eye after being sutured with a 10-0 nylon suture.

The optical central cylinder of this modified KPro is made of PMMA. The anterior-posterior length of the optical cylinder is 3.25 mm, and the overall diameter of its front plate is 5.00 mm, which has a convex curvature. The back diameter of the cylinder is 3.25 mm and is flat. The back plate is made of titanium with a diameter of 8.50 to 9.00 mm, with eight large holes and 0.50 mm thickness. The titanium plate has a slit opening for temporary widening and subsequent closing that surrounds the central cylinder. The power of the central optical cylinder is constantly 50 diopters [Figure 1, Table 1].

### Surgical Technique

All surgical procedures were performed by one surgeon (FK). Under general anesthesia, the patient's eye was first prepared and draped for surgery under sterile conditions. The conjunctival fornices were irrigated with a 5% povidone-iodine solution. A Flieringa ring was placed and sutured using 7-0 silk thread to the episclera at a distance of 2–3 mm from the limbus. First, the donor corneal button, provided by the Central Eye Bank of Iran, was prepared. It featured an appropriate quality and contained more than 2500 endothelial cells. The donor was punched from the endothelial side using an 8.50–8.75 mm Barron corneal donor punch (Katena, NJ, USA). After the exact centration of the donor corneal button, it was punched with a 3-mm skin punch. The ORC-KPro was then assembled as previously described.^[6]^ Briefly, the cylinder (front plate) was placed on a pre-prepared adhesive. Then, the corneal button was placed on the front plate with the endothelium facing upward. In the next step, the back plate was pressed down around the posterior central 3-mm optical cylinder, using a manufactured cylinder with continuous and relatively high pressure, until a “snap” was heard. There was no need for a locking ring or screw in this model. The recipient cornea was trephined with a Hessburg-Barron vacuum trephine (Katena, NJ, USA) with a diameter of 8.25–8.5 mm, depending on the patient's corneal size. The recipient cornea was completely removed. Then, total iridectomy, lens extraction, and anterior vitrectomy were performed, if applicable. The donor corneal button–KPro complex was placed on the eye and sutured to the 0-10 nylon interrupted sutures [Figure 1]. All suture knots were buried. After assurance that there was no leakage, cefazolin and betamethasone were injected subconjunctival. A subtenon injection of 40 mg triamcinolone was performed and an extended-wear bandage contact lens (BCL) (Balafilcone, Pure Vision, Bausch & Lomb, New York, USA) was placed on the eye. Punctal occlusion was performed for all patients. Lateral tarsorrhaphy was performed when it was predicted that the BCL would not be held on the eyes.

### Patients' Follow-up

Follow-up examinations were performed by KH and FK. In the postoperative regimen, levofloxacin drops and preservative-free steroids (NPS, manufactured by the Ophthalmic Research Center, compounding pharmacy) were administered to the patients every 6 hours. The patients received oral acetazolamide (250 mg every 6 hours) after the surgery, especially if they had a history of glaucoma, in order to prevent the spike of intraocular pressure (IOP) due to the retained viscoelastic material. Levofloxacin was reduced to twice a day and continued for one month. Topical steroids were tapered over two months. In case of no systemic contraindication, oral prednisone (1 mg/kg) was initiated and tapered to discontinue in two weeks. Oral ciprofloxacin (500 mg) was prescribed twice a day and continued for five days to prevent endophthalmitis. Systemic anti-glaucoma medication (acetazolamide 250 mg, 3 to 4 times a day) was also initiated and continued due to the impossibility of accurately measuring the IOP. The patients were followed up weekly in the first postoperative month, every two weeks after one month, and monthly after three months. The BCL was also replaced monthly.

In each visit, several assessments were undertaken, including slit-lamp examination, subjective refraction, BCVA, IOP (checked by finger palpation), and fundoscopy (mostly visible optic disc and posterior pole), and the patients' data were recorded.

### Statistical Analysis

Mean and standard deviation were used to describe the data. All data were analyzed using SPSS (version 25, IBM, Armonk, New York).

##  RESULTS

This study was conducted on 12 eyes of 12 patients with an average age of 45.9 ± 16.8 (range, 19 to 70) years. Eleven patients were male. The KPro indication was corneal blindness caused by chemical burns in nine patients (75%). Three patients (25%) were selected for KPro due to lack of an indication for standard corneal grafts, lack of qualification for stem cell transplantation and postoperative immunosuppressive treatment, and failure of multiple previous corneal grafts. There was no intra- or early postoperative complication. The average follow-up time was 12.6 ± 3.5 (range, 6 to 18) months.

### Anatomical Outcome

**Figure 2:**
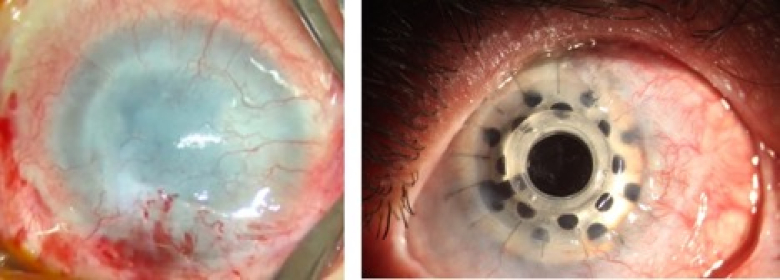
Patient number 3 with chemical burns and a history of multiple surgical procedures of stem cell transplant and corneal transplant before the surgery (left side) and one month after the surgery (right side).

During the follow-up period, there was no leakage (surrounding lenticle–donor cornea or donor–recipient cornea interface) in any of the patients, and anatomical success was achieved in all patients [Table 2]. The representative pre- and postoperative images of a patient are demonstrated in Figure 2.

### Visual Outcome

The preoperative BCVA was light perception in 10 patients and hand motion in 1 patient. Except for one patient who was diagnosed with grade C proliferative vitreoretinopathy (PVR) during the operation, the vision of other patients (91.6%) improved after surgery. BCVA increased to 20/200 in four patients and to 20/80 in one patient. The remaining six patients' vision was counting fingers. All six patients with increased vision to the extent of counting fingers were later diagnosed with optic nerve atrophy during retinal examination [Table 2]. Vision improvement was sustained in seven patients (58.3%) until the last follow-up; the average follow-up period of these seven patients was 12.1 ± 3.9 months.

### Retroprosthetic Membrane (RPM)

The RPM was formed in two patients (18.1%) after six months. The formed membrane was disrupted using Nd-YAG laser in one patient. In the other patient, there was no need to treat the membrane until the last follow-up, when it was stabilized by increasing the steroid frequency.

### Corneal Melting

One of the patients had extensive eyelid irregularity due to extensive burns and a history of eyelid reconstruction multiple times. Due to the lid defects in the patient's lower eyelid and the subsequent exposure, the sclera and the lower part of the cornea developed melting five months post-operation. The patient underwent a corneoscleral patch graft and repeated eyelid reconstruction. The patient was followed up for nine months. In the last follow-up of this patient, the exposure improved and there was no more sign of leakage or melting in the previous site. During the follow-up, lateral tarsorrhaphy was repeated to further protect the melted area.

### Glaucoma 

Ten out of the twelve patients (83.3%) were treated with two antiglaucoma medications (dorzolamide and timolol eye drops) before surgery. The IOP of three patients (25%) was greater than 20 mmHg by palpation, however, in two patients, it decreased to the acceptable range ( < 20–25 mmHG) following an increase in topical medications. Three patients underwent Ahmed glaucoma valve implantation nine months post-operation. During this procedure, the tube was placed in the superior-temporal quadrant, and the IOP was controlled over the follow-up period.

**Table 1:** Patients' characteristics and clinical outcome.


**No.**	**Diagnosis**	**Age (yr)**	**Follow-up duration**	**Anatomical success**	**Preoperative BCVA**	**Postoperative BCVA**	**Final visit BCVA**	**Complication**
1	Burn	19	18	Yes	LP	20/100	HM	RRD and extrusion of KPRO
2	Burn	42	16	Yes	LP	CF 1 m	CF 1 m	RPM
3	Burn	34	17	Yes	LP	CF 1 m	CF 1 m	Optic atrophy
4	Burn	52	7	Yes	LP	20/80	LP	Endophthalmitis
5	Failed graft	70	12	Yes	LP	20/200	20/200	Optic atrophy
6	Failed graft	65	12	Yes	LP	CF 1 m	CF 1m	Optic atrophy
7	Burn	30	15	Yes	HM	20/100	20/200	KPro deposition
8	Burn	31	9	Yes	LP	LP	LP	Intraoperative inoperable RRD
9	Burn	32	12	Yes	LP	20/200	20/200	Scleral melting and globe perforation
10	Failed graft (HSV)	67	12	Yes	LP	CF 2 m	CF 2 m	–
11	Chemical burn	56	9	Yes	LP	CF 4 m	CF 4 M	Optic atrophy
12	Chemical burn	53	6	Yes	LP	20/200	CF 2 M	Endophthalmitis
	
	
BCVA, best-corrected visual acuity; HSV, herpes simplex virus; RRD, rhegmatogenes retinal detachment; CF, counting finger; LP, light perception; HM, hand motion								

**Figure 3:**
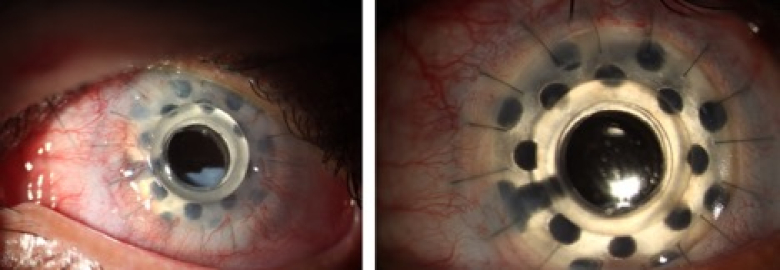
Occurrence of retroprosthetic membrane in a patient with chemical burns who underwent ORC-KPro (left side), and the same patient after removing the membrane with YAG laser.

### Vitreoretinal Complications

One patient underwent retinal surgery due to total retinal detachment six months after the initial surgery, however, BCVA did not improve after the surgery. Two patients (16.7%) underwent surgery due to endophthalmitis, which occurred two and nine months after KPro implantation, respectively. One of these patients developed extensive necrosis of the retinal tissue during the operation. One week after retinal surgery, KPro was explanted and the patient underwent a tectonic graft due to corneal ulceration and melting. The bacterial culture revealed coagulase-negative staphylococcus, and the patient's vision did not improve after two procedures.

In another patient, after endophthalmitis surgery, the patient's vision increased from light perception to counting fingers, but it did not reach the level of vision after the initial surgery (20/200). The result of the culture was coagulase-negative staphylococcus.

In the fourth month after surgery, one of the patients experienced decreased vision (hand motion) and vitreous condensations in the ultrasound B-scan. However, it improved two days after the intravitreal injection of vancomycin, ceftazidime, and intravenous systemic antibiotics, in addition to the initiation of oral steroids. In this patient, the culture result was negative and the diagnosis was sterile vitritis.

Vitreous hemorrhage was seen in two patients in the first month after surgery which was absorbed after two weeks through conservative management and thereby, the vision improved significantly.

##  DISCUSSION

The results of the current study showed that the ORC-KPro was anatomically successful in all cases (12 eyes so far) with short follow-up. No leakage was observed in any of the eyes during an average of 12.1 months. After the implantation of the ORC-KPro, the BCVA improved in all eyes except for one. This lack of improvement was caused by an old and extensive retinal detachment complicated by PVR which had not been detectable in the B-scan sonography before KPro implantation due to the presence of silicone oil in the vitreous cavity.

Boston KPro was introduced and developed by Claes Dohlman in 1965. During the past decades, its design has been completed and refined and, therefore, its indications of use have expanded. Boston KPro has been invented for those cases of corneal blindness where “standard” corneal transplantation involves a high risk for the recipient's cornea or multiple previous transplantations have failed. KPro is used as the last resort to improve vision in patients with corneal blindness.^[16,17]^

Due to the limited availability of the original Boston KPro in Iran, a new KPro, namely ORC-KPro, was designed and developed in the Ophthalmic Research Center, affiliated with Shahid Beheshti University of Medical Sciences. In the current study, as the pilot stage for this type of prosthesis, we examined patients with end-stage corneal blindness. Most of our cohort had reached the advanced stage of the underlying disease, such that a significant percentage of patients showed optic atrophy after the ORC-KPro operation.

The clinical outcome of Boston type 1 KPro highly depends on the indication of its use. It yields the worst results when indicated in patients with Stevens-Johnson syndrome or mucous membrane pemphigoid.^[2,11]^ This is because of chronic inflammation and lack of tear film in these patients, which can lead to donor corneal melting and/or KPro extrusion. ${\;}^{\left[2,11,12,18\right]}$ In the present study, the main indication for KPro implantation was chemical burns. The survival outcomes of ORC-KPro and the corresponding vision we observed in the patients are comparable to the results of Boston type 1 KPro in patients with the same indication.^[2,11][12][18]^ Ocular burn is a relatively high-risk indication for Boston type 1 KPro^[28]^ due to concomitant conditions such as glaucoma, compromised ocular surface, symblepharon, and ongoing chronic inflammation in these patients. However, some studies have reported promising long-term results. In one multicenter study on Boston type 1 KPro, 19 patients with chemical burns were assessed: 17 patients achieved visual acuity > 20/200 and 16 patients maintained BCVA > 20/200.^[29]^ Salvador-Culla et al also reported excellent retention and favorable visual acuity as the long-term outcomes of Boston type 1 KPro in patients with ocular burn.^[30]^

The visual outcome and the complications of Boston type 1 KPro have been reported in many recent studies. Some of the main causes of vision reduction include glaucoma and its progression, infectious endophthalmitis, and RPM.^[5]^ The more advanced stage of the disease in the present study may partly explain the lower level of vision stability. Comparing preoperative visual acuity, the percentage of patients with glaucoma, and the number of previous surgical procedures in the current study with those involving long-term follow-ups reveals that our patients underwent surgery in a more advanced stage of the disease.^[5]^ Also, the sample size in the current study was small, and our main purpose was to investigate the surgical anatomical success and the weak points of ORC-KPro implantation, which comprised the pivotal stage of this study.

In the current study, the percentage of patients with treated glaucoma before KPro implantation was 83.3% (9/12), and high IOP occurred in three patients after the procedure. Glaucoma is the first cause of irreversible vision loss after KPro implantation. The rate of glaucoma development or deterioration varies widely, ranging from 9% to 82% in different studies.^[20,21]^ Some of the causes and risk factors for glaucoma development include altered structure of the iridocorneal angle due to prosthesis placement, involvement of the iridocorneal angle by the RPM, and destruction of collecting channels by chemical burn (as the KPro indication). Newer insights about the role of inflammatory cytokines in secondary retinal damage after injury or KPro surgery reported by the Boston KPro study group may offer an adjunct therapy for retinal neuroprotection in the future.

The most controllable risk factor in patients with glaucoma is IOP, which cannot be accurately measured after KPro implantation.^[22]^ To solve this problem, some researchers recently integrated a pressure sensor directly on a prototype Boston KPro device.^[23]^ Also, in some centers, it is recommended to perform shunt surgery alongside KPro surgery.^[23]^

RPM was observed in 16.6% (2/12) of cases during an average follow-up of 12 months. The reason for this lower incidence compared to similar studies can be attributed to the type and size of the posterior plate in the ORC-KPro design, the absence of visible inflammation at the time of surgery, and the short follow-up period. The reported risk factors for RPM formation include infectious etiologies, keratitis, the presence of active inflammation, simultaneous surgery with other surgical procedures, and retinal tears.^[24]^ It has also been speculated that RPM develops less frequently in titanium back plates because of its material, design, and larger size.^[25]^

Infectious endophthalmitis was observed in 16.6% (2/12) of patients. Endophthalmitis can occur in patients who undergo KPro surgery at any time post-operation. For this reason, patients must receive long-life prophylaxis with new-generation fluoroquinolone antibiotic drops and vancomycin.^[10,26]^ The most common causes of endophthalmitis in patients undergoing KPro are gram-positive organisms (such as staphylococcus and streptococci) that are present as normal flora on the ocular surface. It seems that the common mechanism of endophthalmitis in these patients is the entry of ocular surface microorganisms through the donor cornea-KPro junction, which is not fully integrated with the surrounding corneal tissue and, therefore, does not serve as a perfect barrier.^[27]^ Endophthalmitis in these patients can manifest as calm eye appearance and vision loss. Only during an examination can one notice the obscuration of the retinal view, loss of red reflex, and opacifications in the vitreous cavity on the B-scan.

The limitations of the current study include a small sample size, the absence of a control group, and a short-term follow-up. Most of our patients had chemical burns, and the results of KPro in this group cannot be generalized to all patients requiring KPro.

In summary, the current study showed that the use of ORC-KPro, manufactured by the Ophthalmic Research Center of Shahid Beheshti University of Medical Sciences, in patients with corneal blindness has favorable anatomical success in short-term follow-up. It is recommended (1) to continue this study and design a randomized controlled trial to compare ORC-KPro with the original Boston KPro (when available), (2) to increase the sample size to consider patients with various types of ocular surface diseases, and (3) to implement longer follow-up periods to provide a better and more accurate picture regarding the safety and efficacy of ORC-KPro.

### Financial Support and Sponsorship

None.

### Conflicts of Interest

None.
